# Calprotectin (S100A8/A9) has the strongest association with ultrasound-detected synovitis and predicts response to biologic treatment: results from a longitudinal study of patients with established rheumatoid arthritis

**DOI:** 10.1186/s13075-016-1201-0

**Published:** 2017-01-12

**Authors:** Hilde Haugedal Nordal, Karl Albert Brokstad, Magne Solheim, Anne-Kristine Halse, Tore K. Kvien, Hilde Berner Hammer

**Affiliations:** 1 Broegelmann Research Laboratory, Department of Clinical Science, University of Bergen, Haukeland University Hospital, The Laboratory Building, 5th floor, Jonas Lies vei 87, N-5021 Bergen, Norway; 2 Department of Rheumatology, Haukeland University Hospital, Bergen, Norway; 3 Department of Clinical Science, University of Bergen, Bergen, Norway; 4 Department of Rheumatology, Diakonhjemmet Hospital, Oslo, Norway

**Keywords:** Rheumatoid arthritis, Biomarkers, Calprotectin, S100A12, Interleukin 6, Vascular endothelial growth factor, Ultrasound

## Abstract

**Background:**

Calprotectin (S100A8/A9 or MRP8/14) and S100A12 (leukocyte-derived proteins), interleukin 6 (IL-6) and vascular endothelial growth factor (VEGF) are markers of inflammation and angiogenesis. Ultrasound (US) is sensitive for detection of greyscale synovitis and power Doppler (PD) vascularization. The objective of the present study was to explore the associations between calprotectin, S100A12, IL-6, VEGF, erythrocyte sedimentation rate, C-reactive protein and a comprehensive US assessment in patients with rheumatoid arthritis (RA) starting biologic disease-modifying anti-rheumatic drug (bDMARD) treatment.

**Methods:**

A total of 141 patients with RA were assessed by US, clinical examination and biomarker levels at baseline and at 1, 2, 3, 6 and 12 months after initiation of bDMARDs. US assessment of 36 joints and 4 tendon sheaths were scored semi-quantitatively (0–3 scale). European League Against Rheumatism (EULAR) response was calculated. Statistical assessments performed to explore the associations between biomarkers and US sum scores included Spearman’s rank correlation analysis as well as linear and linear mixed model regression analyses.

**Results:**

Calprotectin showed the overall strongest correlations with both US sum scores (*r*_s_ = 0.25–0.62) and swollen joint counts (of 32) (*r*_s_ = 0.24–0.47) (*p* < 0.05 at all examinations). An association with US sum scores remained after we adjusted for age, sex, disease duration and all the other markers in a regression analysis at baseline. Decreased calprotectin at the first month was predictive of both EULAR response (*p* ≤ 0.001) and decreased sum PD scores at 3, 6 and 12 months (*p* ≤ 0.05).

**Conclusions:**

Calprotectin had the highest association with US synovitis and predicted treatment response. It may thus be considered as a marker for evaluating inflammation and responsiveness in patients with RA on bDMARD treatment.

**Trial registration:**

Australian New Zealand Clinical Trials Registry (ANZCTR) identifier: ACTRN12610000284066. Registered on 8 April 2010 (retrospectively registered).

**Electronic supplementary material:**

The online version of this article (doi:10.1186/s13075-016-1201-0) contains supplementary material, which is available to authorized users.

## Background

Rheumatoid arthritis (RA) is an autoimmune disease with a complex pathogenesis involving innate and adaptive immunity as well as angiogenesis [1, 2]. Biomarkers can reflect these reactions and provide support in diagnostic and prognostic evaluations. Despite intensive research, only C-reactive protein (CRP) and erythrocyte sedimentation rate (ESR) are regularly used as inflammatory biomarkers in the clinics.

Calprotectin is a heterocomplex of the S100 proteins S100A8 and S100A9 (also called *myeloid-related protein 8* [*MRP8*] and *MRP14*), found mainly in circulating neutrophils and monocytes and in macrophages in RA synovial tissue [3, 4]. Up to 60% of the cytosolic protein content in granulocytes is reported to be calprotectin [5]. S100A12 is another inflammation-associated S100 protein, expressed predominantly in neutrophils and comprising about 5% of the protein content in the cytosol [6]. Calprotectin and S100A12 are pro-inflammatory proteins. By their putative binding to Toll-like receptor 4 or receptor for advanced glycation endproducts on granulocytes, monocytes, or endothelial cells, the S100 proteins have the potential to induce production of important cytokines in RA, such as interleukin (IL)-1β, IL-6 and tumour necrosis factor, as well as intracellular and vascular cell adhesion molecules, via the nuclear factor-κB pathway [7]. In addition, calprotectin and S100A12 can be chemoattractants for neutrophils and monocytes/mast cells, respectively [7] and can thus in several ways contribute to recruitment of leukocytes to inflammatory sites. Both proteins have﻿﻿, when measured ﻿in plasma or serum, been repeatedly reported to be associated with clinical disease activity in RA [8–18]. In addition, calprotectin was associated with radiographic damage in a cross-sectional study and predicted radiographic progression in a longitudinal study [11, 19], whereas S100A12 has shown a weak association with radiographic damage in a small longitudinal study [17].

IL-6 is a cytokine that stimulates hepatocytes (production of CRP and other acute-phase proteins), B cells (maturation, antibody production), T cells (Th17 differentiation), neutrophils (activation and migration), osteoclasts (maturation) and vascular endothelial growth factor (VEGF) [20]. IL-6 has been associated with disease activity and radiographic progression [21]. VEGF is an important inducer of angiogenesis as well as a vascular permeability factor [2]. It was found to be associated with disease activity [22, 23] and with radiographic destruction in patients with RA [24].

 Ultrasound (US) is a sensitive and validated imaging technique for evaluation of synovitis in patients with RA [25]. The inflammation is usually scored semi-quantitatively for both the amount of synovitis (greyscale [GS]) and the degree of vascularization (power Doppler [PD]). High reliability has been demonstrated for scoring of synovitis of a large number of joints in patients with RA by use of a US atlas as a reference [26].

To date, there have been two small longitudinal studies and two small cross-sectional studies on the correlations between the two S100 proteins and US findings [16, 27–29]. IL-6 has been shown to have significant correlations with US synovitis in cross-sectional studies of early disease-modifying anti-rheumatic drug (DMARD)-naïve patients with RA [30] and with PD score in patients with established RA treated with non-biologic DMARDs [31], respectively. Being a marker of angiogenesis, VEGF may be associated with Doppler activity in inflamed RA joints. However, so far, there is no agreement on the association between VEGF and PD [32–38].

Markers such as ESR and CRP are frequently used but may not always reflect the ongoing synovitis. Thus, there is a need for new biomarkers that have stronger associations with inflammation and angiogenesis in patients with RA. The main objective of the present study was to explore associations between inflammatory/angiogenic markers (calprotectin, S100A12, IL-6 and VEGF) and a comprehensive US examination, as well as clinical assessments, in a longitudinal study of patients with established RA starting biologic disease-modifying anti-rheumatic drug (bDMARD) treatment.

## Methods

### Patients and control subjects

A total of 141 patients with RA, all meeting the 1987 revised American Rheumatism Association classification criteria [39], were consecutively included at an outpatient rheumatology clinic in the period from January 2010 to June 2013 when starting a bDMARD. A total of 45.4% started with their first bDMARD, 30.5% with their second bDMARD, and 24.1% had used up to three to five bDMARDs previously. All patients continued their bDMARD during the study, and they were assessed at baseline and at 1, 2, 3, 6 and 12 months. Age- and sex-matched healthy control subjects (blood donors) recruited from Haukeland University Hospital, representing the same ethnic origin as the patients, were included for obtaining normal levels of S100A12, IL-6 and VEGF in serum (Table 1), whereas normal levels of calprotectin in ethylenediaminetetraacetic acid (EDTA) plasma were given by the manufacturer CALPRO AS, Lysaker, Norway (based on levels from blood donors at Ullevål University Hospital).

**Table 1:** Baseline patient and control characteristics

	Patients (*n* = 141)	Control subjects (*n* = 141)	*p* Values
Age, years	54 (45–61)	54 (45–61)	0.96
Women	114 (80.9)	114 (80.9)	1.0
Disease duration, years	6.8 (3.0–14.9)		
RF-positive	100 (70.9)		
Anti-CCP-positive	109 (77.3)		
Using methotrexate	109 (77.3)		
Using prednisolone	78 (55.3)		
Biologic DMARD at inclusion			
Infliximab	13 (9.2)		
Etanercept	56 (39.7)		
Adalimumab	6 (4.3)		
Golimumab	8 (5.7)		
Certolizumab	11 (7.8)		
Rituximab	32 (22.7)		
Abatacept	3 (2.1)		
Tocilizumab	12 (8.5)		
Patient’s global VAS, 0–100	46 (20–67)		
Assessor’s global VAS, 0–100	27 (18–38)		
Number of tender joints (of 32)	4 (2–11)		
Number of swollen joints (of 32)	6 (3–11)		
DAS28(ESR)	4.4 (1.4)^a^		
Sum GS US score	27 (17–43)		
Sum PD US score	11 (4–24)		
ESR, mm/h	22 (11–35)		
CRP, mg/L	6 (2–12)		
Calprotectin, ng/ml	1149 (698–1949)	560 (412–796)^b^	<0.001
S100A12, ng/ml	275 (142–463)	129 (75–222)	<0.001
IL-6, pg/ml	5.2 (2.1–10.8)	0.9 (0.2–1.6)	<0.001
VEGF, pg/ml	80.8 (42.8–161.8)	67.5 (45.6–108.0)	<0.08

*Abbreviations*: *RF* Rheumatoid factor, *anti-CCP* Anti-cyclic citrullinated peptide, *DMARD* Disease-modifying anti-rheumatic drug, *VAS* Visual analogue scale, *DAS28* Disease Activity Score in 28 joints, *ESR* Erythrocyte sedimentation rate, *GS* Greyscale, *US* Ultrasound, *PD* Power Doppler, *VEGF* Vascular endothelial growth factor, *IL-6* Interleukin 6

Data are median (interquartile range) for continuous variables and number (percent) for categorical variables

^a^Mean (±SD)

^b^Control subjects are healthy blood donors (50 men and 50 women) analysed by the manufacturer ﻿CALPRO AS, Lysaker, Norway

### Clinical disease activity

The 28-joint examination of tender and swollen joints [40], with the addition of bilateral ankle and metatarsophalangeal (MTP) joints 1–5 (the MTP joints evaluated as one joint) (i.e., a total of 32 joints), was performed by two trained and experienced study nurses blinded to the US results. The assessor’s (study nurse’s) global evaluation of disease activity, as well as the patient’s global assessment of disease activity and pain, were recorded on 0- to 100-mm visual analogue scales. The Disease Activity Score in 28 joints including ESR (DAS28) [40] was calculated, and improvement of disease activity from baseline was recorded according to the European League Against Rheumatism (EULAR) response criteria [41].

### Ultrasound

An experienced sonographer (HBH) performed all the US examinations (using an Antares Excellence version 5- to 13-MHz probe [Siemens Healthcare, Erlangen, Germany], optimized for PD with pulse repetition frequency 391 Hz, and no updates during the study) and had no access to the clinical assessments and biomarkers from the same time point or to previous US results. GS and PD were scored semi-quantitatively on a 4-point scale (0 = no, 1 = minor, 2 = moderate, 3 = major presence) of 36 joints (bilateral wrist, comprising radiocarpal [RC], midcarpal [MC] and radioulnar [RU] joints; metacarpophalangeal [MCP] joints 1–5; proximal interphalangeal (PIP) joints 2 and 3; elbow; knee; ankle [tibiotalar]; and MTP1–5) and 4 tendon sheaths (extensor carpi ulnaris [ECU] and tibialis posterior [TP]) [26]. The size of the joint may influence the burden of synovitis. Thus, weighting of joints according to the method of Lansbury [42] was also included when exploring associations. (US scores of MCP, PIP and MTP joints were multiplied by 1; RC, MC and RU joints were multiplied by 2; ECU and TP sheaths were multiplied by 2; elbow was multiplied by 12; knee was multiplied by 25; and the ankle was multiplied by 9.)

### Assessment of biomarkers

Rheumatoid factor, anti-cyclic citrullinated peptides, CRP and ESR were analysed by routine in-house methodology. The samples were treated as uniformly as possible, centrifuged within 1 h after sampling, and plasma/serum was removed and put in the freezer as soon as possible and carefully thawed in a refrigerator overnight before the analysis. All samples from the same patient (and respective control subject) were thawed at the same time for the analyses of the biomarkers. Laboratory personnel (KAB, MS and HHN) were blinded to the results of the clinical, US and laboratory assessments. Calprotectin was analysed in EDTA-plasma by a commercial enzyme-linked immunosorbent assay (ELISA) (CALPRO AS, Lysaker, Norway), and S100A12 was analysed in serum by ELISA (CycLex Co., Nagano, Japan), both according to the instructions of the manufacturers. The plates were read by an Emax plate reader with Softmax Pro software version 5.4 (Molecular Devices, Sunnyvale, CA, USA). IL-6 and VEGF were analysed in serum by a premixed multi-analyte kit (R&D Systems, Minneapolis, MN, USA), following the instructions of the manufacturer, and the plates were read on a Luminex 100 system (Luminex Corp., Austin, TX, USA) with use of STarStation version 3.0 software (Applied Cytometry Systems, Dinnington, UK).

### Statistics

The Mann-Whitney *U* test or the Wilcoxon signed-rank test was used to evaluate differences between and within groups and to study changes from baseline, respectively. Correlations were explored by Spearman’s rank correlation analyses. Linear regression was used for further studies of associations. In a first step, we adjusted for age, sex and disease duration, and then all the markers were included in a second step. To predict change in US sum scores, a linear mixed model regression analysis was used. All markers had right-skewed distributions and were log_2_-transformed prior to inclusion in the regression models. IBM SPSS Statistics version 23 (IBM, Armonk, NY, USA), R version 3.2.3 for Windows (http://www.r-project.org) and Prism version 5 (GraphPad Software, La Jolla, CA, USA) software were used for the statistical analyses and to create figures. All tests for significance were two-sided, and *p* < 0.05 was considered significant. Plasma/serum samples or clinical data were not available for 31 (3.7%) of 846 examination time points. The missing data were replaced by the last observation carried forward.

## Results

**Fig. 1:**
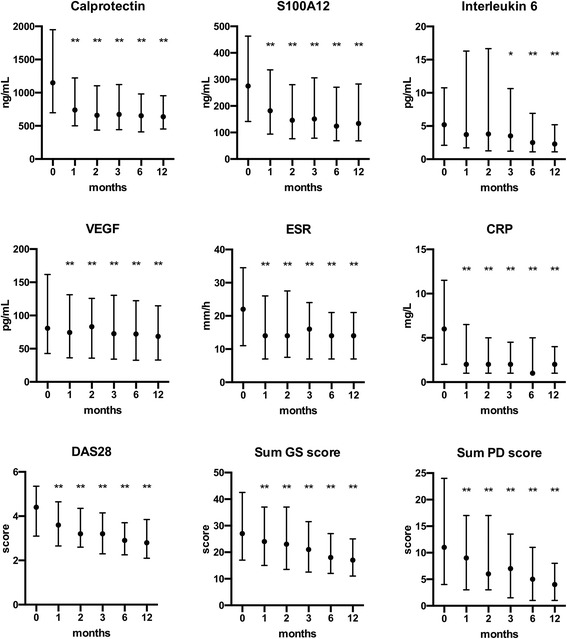
Median levels (error bars = interquartile range) of inflammatory markers, Disease Activity Score in 28 joints (DAS28) and ultrasound sum score (greyscale [GS] and power Doppler [PD]) at baseline and during follow-up. * *p* < 0.05, ** *p* < 0.001 reduction from baseline. *CRP* C-reactive protein, *ESR* Erythrocyte sedimentation rate, *VEGF* Vascular endothelial growth factor

Baseline levels of calprotectin, S100A12 and IL-6 were significantly higher in patients than in control subjects, but this was not shown for VEGF (Table 1). DAS28, the US sum scores and all the biomarkers (except IL-6) decreased significantly from baseline (Fig. 1). However, IL-6 was significantly elevated after baseline in sera of patients starting with tocilizumab (*n* = 12, 8.5%) (Additional file 1: Figure S1). When the patients on tocilizumab were omitted from the calculation, IL-6 showed a significant decrease (*p* < 0.001) from baseline at all time points, except at 2 months (*p* = 0.06) (results not shown).

Of the biomarkers, calprotectin had the strongest and most consistent associations with clinical variables and US sum scores in correlation analyses at baseline (Table 2), and there were comparable correlation coefficients during follow-up (Additional file 2: Table S1). We also analysed the two largest subgroups of patients on bDMARDs (i.e., etanercept [*n* = 56] and rituximab [*n* = 32]). Calprotectin was the marker most strongly associated with US sum scores in these subgroups. In patients on etanercept, calprotectin had median (range) correlation coefficients of 0.46 (0.13–0.52) with GS sum score and 0.43 (0.15–0.58) with PD sum score (*p* = ns to <0.001). In rituximab users, calprotectin had correlation coefficients of 0.58 (0.39–0.73) with GS sum score and 0.53 (0.41–0.70) with PD sum score (*p* = 0.03 to <0.001). The correlations between calprotectin and US sum scores weighted according to the method of Lansbury were not much different from, and not consistently stronger than, correlations between calprotectin and the original US sum scores (Additional file 3: Table S2). Patients on tocilizumab (*n* = 12, 8.5%) had overall higher correlations between calprotectin and US sum scores compared with ESR and CRP during follow-up, although these correlations were not always significant (Additional file 4: Table S3).

**Table 2:** Spearman’s rank correlation coefficients between biomarkers and ultrasound sum scores and clinical parameters at baseline

	Sum GS score	Sum PD score	Assessor’s global VAS	DAS28	Swollen joint count (of 32)	Tender joint count (of 32)	Patient’s global VAS	Joint pain VAS
Calprotectin	0.59^a^	0.62^a^	0.60^a^	0.49^a^	0.47^a^	0.17^b^	0.27^a^	0.32^a^
S100A12	0.39^a^	0.42^a^	0.44^a^	0.35^a^	0.35^a^	0.14	0.22^b^	0.23^b^
IL-6	0.42^a^	0.49^a^	0.53^a^	0.39^a^	0.41^a^	0.09	0.20^b^	0.24^b^
VEGF	0.20^b^	0.18^b^	0.14	0.10	0.13	−0.03	0.15	0.21^b^
ESR	0.19^b^	0.30^a^	0.46^a^	0.67^a^	0.22^b^	0.27^a^	0.28^a^	0.33^a^
CRP	0.41^a^	0.47^a^	0.46^a^	0.47^a^	0.30^a^	0.15	0.18^b^	0.24^b^

*Abbreviations*: *Sum GS score* Sum of greyscale scores on a 0–3 scale of 36 joints and four tendon sheaths, *Sum PD score* Sum power Doppler scores on a 0–3 scale of 36 joints and 4 tendon sheaths, *VAS* Visual analogue scale, *DAS28* Disease Activity Score in 28 joints (including erythrocyte sedimentation rate), *VEGF* Vascular endothelial growth factor, *IL-6* Interleukin 6, *ESR* Erythrocyte sedimentation rate, *CRP* C-reactive protein

^a^ *p* < 0.05

^b^ *p* ≤ 0.001

**Fig. 2:**
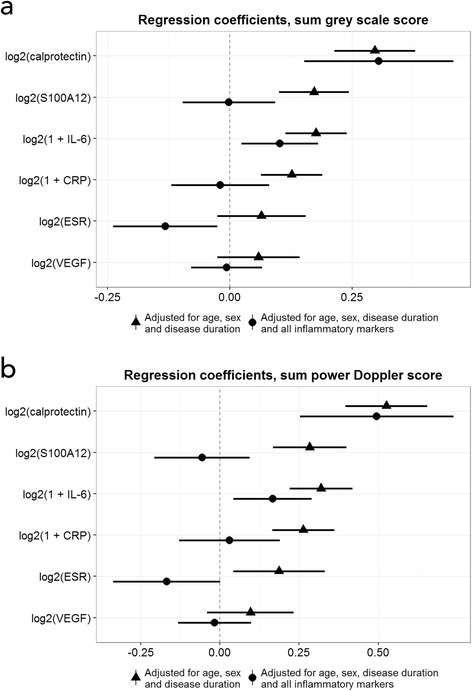
Linear regression model, baseline. Coefficients with 95% confidence intervals with (**a**) greyscale sum score and (**b**) power Doppler sum score as dependant variables. *IL-6* Interleukin 6, *CRP* C-reactive protein, *ESR* Erythrocyte sedimentation rate, *VEGF* Vascular endothelial growth factor

Cross-sectional linear regression analysis at baseline is presented in Fig. 2. Calprotectin could best explain the US scores after adjusting for age, sex and disease duration, as well as after additional adjustment for all the other markers.

The influence of prednisolone use was explored by assessing differences between patients using or not using prednisolone at baseline. Patients on prednisolone at baseline (*n* = 78 [55.3%], median [range] dose 7.5 [2.5–25] mg) had higher levels of calprotectin (*p* = 0.006), GS sum score (*p* = 0.02) and number of swollen joints (*p* = 0.02), whereas no differences were found between the groups for any of the other markers or clinical variables.

To explore whether the biomarkers had normal levels in patients without active synovitis, we analysed the biomarkers in patients with a PD sum score of 0 after 12 months (*n* = 30) and compared them with the levels found in healthy control subjects (Additional file 5: Figure S2). For calprotectin, S100A12 and VEGF, there was no difference between the patients and the control subjects, whereas IL-6 was higher in the patients (although patients on tocilizumab were excluded).

Changes (∆) in the biomarkers were calculated as the differences from baseline to 1, 2, 3, 6 and 12 months. Except for the association between ∆ESR and ∆DAS28, ∆calprotectin had the overall strongest associations with the corresponding changes in US sum scores and DAS28 (Additional file 6: Table S4).

The median (interquartile range) levels of the biomarkers in EULAR good, moderate and non-responders at 3, 6 and 12 months are shown in Additional file 7: Table S5. The largest differences between the groups were found for ESR and CRP. EULAR responders (good and moderate) at 3 months had significantly higher baseline values of calprotectin (*p* = 0.001), S100A12 (*p* = 0.02), IL-6 (*p* = 0.02) and CRP (*p* = 0.003) than non-responders. Although calprotectin showed a trend (*p* = 0.08), EULAR responders at 6 months did not have significantly higher values of any of the markers at baseline. For EULAR responders after 12 months, only calprotectin (*p* = 0.03) had significantly higher baseline values. In addition, EULAR responders recorded at 3-, 6- and 12-month follow-up had significantly higher ∆calprotectin during the first month (*p* ≤ 0.001 for all time points), whereas such a strong difference was not found for the other markers. (ESR was not included in this calculation, because it is a part of the EULAR response criteria.)

In the linear mixed model analysis, there was a trend for ∆calprotectin during the first month to predict change in GS sum scores after 3 months (*p* = 0.09). However, significant prediction was found after 6 months (*p* = 0.03) but not after 12 months (*p* = 0.20). ∆ESR did not predict change in GS sum scores, whereas ∆CRP predicted change in GS sum scores after 12 months (*p* = 0.01). ∆Calprotectin during the first month predicted change in PD sum scores at 3, 6 and 12 months (*p* = 0.05, 0.004 and 0.002, respectively). ∆ESR also predicted change in PD sum scores at 3, 6 and 12 months (*p* = 0.01, 0.01 and <0.001, respectively), whereas ∆CRP was predictive of change in PD sum score at 6 months (*p* = 0.03) and 12 months (*p* < 0.001). ∆S100A12, ∆VEGF and ∆IL-6 did not significantly predict change in GS or PD sum scores.

## Discussion

In this 1-year follow-up study of 141 patients with RA starting bDMARDs, we found calprotectin to be more strongly associated with US and clinical scores of inflammation than ESR, CRP, S100A12, IL-6 and VEGF. In addition, a higher level of calprotectin was found in EULAR responders, and decrease of calprotectin during the first month predicted reduced PD sum scores at 3, 6 and 12 months.

The present study supports the previous finding of strong associations between calprotectin and US sum scores in a small longitudinal study [27] and two small cross-sectional studies [28, 29]. The association is likely due to calprotectin being found in leukocytes, and, with its impact on immune cells and endothelial cells in the joints, calprotectin may have the ability to reflect the US findings of inflammation. This is also supported by the finding of normal levels of calprotectin in patients with no sign of PD activity, which is in agreement with a recent study in which calprotectin could help identify PD synovitis in patients with DAS28 ≤ 3.2 [29].

There are conflicting results on baseline calprotectin as a predictive marker of response to bDMARDs [13, 17, 43]. High levels of biomarkers at baseline imply a large potential for improvement but might not consistently predict response to treatment. Calprotectin showed a rapid decrease during bDMARD treatment, and ∆calprotectin after 1 month predicted EULAR response. In addition, calprotectin and ESR were the only biomarkers where change during the first month predicted change in PD sum scores. Thus, calprotectin may have a potential for early prediction of response to bDMARD treatment. ∆Calprotectin during the first month was more predictive of change in PD sum score than change in GS sum score. This might be explained by PD detecting arterioles dilated because of chemical and inflammatory mediators locally in the area of synovitis. PD activity is found to diminish rapidly after glucocorticoid injection in a joint, and this is thought to be caused by a reduction of inflammatory mediators causing the arterioles not to be dilated [44], even if they are still present in the synovium. Thus, on one hand, calprotectin may be associated with, and may predict, the PD activity because both calprotectin and PD are reflecting inflammation. The GS findings, on the other hand, represent synovium that may be more or less actively inflamed and may therefore not have the same association with inflammatory markers. As a parallel, in inflammatory bowel diseases, calprotectin levels (in faeces) have been shown to be associated with inflammation and found to be highly useful in the clinics by predicting clinical response and mucosal healing in patients treated with biologics [45].

Tocilizumab suppresses the conventional inflammatory markers. Only a small number of patients used tocilizumab in this study. However, calprotectin was shown to have stronger associations with the US scores than ESR and CRP in these patients. This is supported by a study of 33 patients with RA treated with tocilizumab in whom calprotectin had stronger correlations with composite scores (DAS28, Simplified Disease Activity Index and Clinical Disease Activity Index) and joint counts compared with CRP and ESR [46].

Prednisolone may decrease inflammatory markers, but use of prednisolone did not seem to suppress the levels of calprotectin, supporting the results of a previous study [11]. Thus, in patients on prednisolone, the inflammatory activity in established RA may be better reflected by calprotectin than by the commonly used ESR and CRP.

Associations of S100A12 with clinical variables and US scores were comparable with those of CRP, but lower than for calprotectin. The explanation for the two S100 proteins’ having different reflection of inflammation could in part be the differences in their distribution and amounts in leukocytes. PD may reflect the angiogenesis, and S100A12 is particularly found in perivascular neutrophils of RA synovia [47] and is able to activate endothelial cells [7]. We previously reported associations between S100A12 and PD in a small longitudinal study [16], and the results of the present study strengthen this preliminary finding by showing consistent associations at all time points.

IL-6 was a relatively strong marker of inflammation in this study, probably reflecting its key functions in RA [1, 20]. This is in agreement with previous studies [21, 30, 31]. However, IL-6 levels increase during tocilizumab treatment [48], which was also shown in this study (Additional file 1: Figure S1).

VEGF had the weakest associations with US and clinical variables in the present study. This angiogenic marker was expected to be associated with PD scores. However, there were low correlations between VEGF and PD sum scores. Previous studies have diverged in designs and results [32–38]. Only small groups of patients were included (15–29 patients) in most of the previous studies on VEGF, and different numbers of joints have been assessed (PD in 1–22 joints). In addition, all but one study had a cross-sectional design. Thus, the present large longitudinal study, in which we examined a high number of joints, supports previous studies indicating that VEGF is not a strong marker of active synovitis in patients with RA.

The present study has several strengths. A large number of patients with RA were included; they were assessed six times during 1 year after initiation of treatment with bDMARDs; and the comprehensive US examination was performed by one experienced sonographer. The study was carried out in a regular clinical setting, which may support the external validity of the findings. A limitation may be the relatively low disease activity in spite of including only patients starting a bDMARD, which could have had an impact on the responsiveness of the inflammatory markers. A high number of joints were examined by US, but the inclusion of even more joints might possibly have influenced the results. Weighting of joints in accordance with the Lansbury method did not increase the correlation between US scores and calprotectin. Whether another form of weighting of joints would increase the associations between calprotectin and US pathology remains to be explored.

## Conclusions

Calprotectin was found to be the biomarker with the strongest associations with both US and clinical disease activity assessments during 1 year of follow-up among patients with established RA starting bDMARDs. In addition, reduction of calprotectin after 1 month was predictive of EULAR response and decrease in PD sum scores. Thus, we suggest that calprotectin measured in EDTA-plasma could be a valuable supplement to the conventional inflammatory markers when assessing patients with RA during follow-up of bDMARD treatment.

## Additional files

Additional file 1: Figure S1. Median (error bars = interquartile range) levels of interleukin 6 in 12 patients (8.5%) starting with tocilizumab at baseline. (PDF 29 kb)
Additional file 2: Table S1. Spearman’s rank correlation coefficients (*r*_s_) between biomarkers and US sum scores and clinical parameters during follow-up. (PDF 51 kb)
Additional file 3: Table S2. Spearman’s rank correlation coefficients (*r*_s_) between calprotectin and US sum and Lansbury US sum scores. (PDF 33 kb)
Additional file 4: Table S3. Spearman’s rank correlation coefficients (*r*_s_) between calprotectin/conventional inflammatory markers and sum US scores in patients using tocilizumab (*n* = 12). (PDF 36 kb)
Additional file 5: Figure S2. Median levels with a range of inflammatory markers in control subjects (*n* = 100 for calprotectin; *n* = 141 for S100A12, IL-6 and VEGF) and patients with no PD activity at 12 months (*n* = 30 for calprotectin, S100A12 and VEGF; *n* = 28 for IL-6 [patients on tocilizumab omitted]). (PDF 64 kb)
Additional file 6: Table S4. Spearman’s rank correlation coefficients (*r*_s_) between changes in inflammatory markers and US sum scores/DAS28 from baseline to 1, 2, 3, 6 and 12 months. (PDF 40 kb)
Additional file 7: Table S5. Median (interquartile range) levels of biomarkers in EULAR good, moderate and non-responder groups at 3, 6 and 12 months. (PDF 39 kb)

## Abbreviations

bDMARD: Biologic disease-modifying anti-rheumatic drug
CCP: Cyclic citrullinated peptide
CRP: C-reactive protein
DAS28: Disease Activity Score in 28 joints including erythrocyte sedimentation rate
DMARD: Disease-modifying anti-rheumatic drug
ECU: Extensor carpi ulnaris
EDTA: Ethylenediaminetetraacetic acid
ELISA: Enzyme-linked immunosorbent assay
ESR: Erythrocyte sedimentation rate
EULAR: European League Against Rheumatism
GS: Greyscale
IL: Interleukin
MC: Midcarpal
MCP: Metacarpophalangeal
MRP: Myeloid-related protein
MTP: Metatarsophalangeal
PD: Power Doppler
PIP: Proximal interphalangeal
RA: Rheumatoid arthritis
RC: Radiocarpal
RF: Rheumatoid factor
RU: Radioulnar
TP: Tibialis posterior
US: Ultrasound
VAS: Visual analogue scale
VEGF: Vascular endothelial growth factor
