# Supplementary material for: Calprotectin (S100A8/A9) has the strongest association with ultrasound-detected synovitis and predicts response to biologic treatment: results from a longitudinal study of patients with established rheumatoid arthritis
Source: Arthritis Res Ther. 2017 Jan 12;19:3. doi: 10.1186/s13075-016-1201-0 (PMC5234113; doi:10.1186/s13075-016-1201-0)
Supplement: Additional file 1: Figure S1. — Median (error bars = interquartile range) levels of interleukin 6 in 12 patients (8.5%) starting with tocilizumab at baseline. (PDF 29 kb) [file 13075_2016_1201_MOESM1_ESM.pdf]

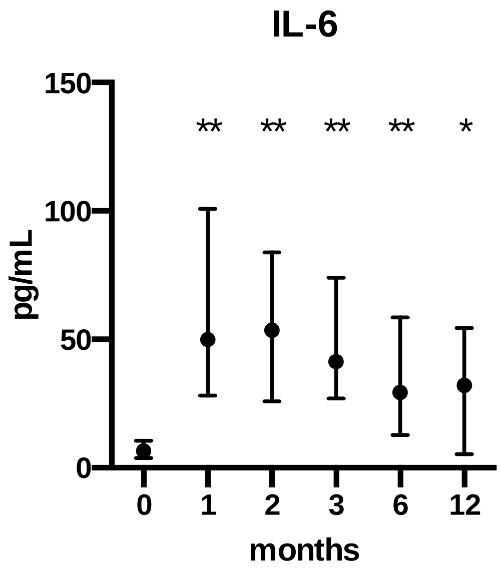

Supplementary figure S1. Median (error bars = interquartile range) levels of interleukin 6 in 12 patients (8.5%) starting with tocilizumab at baseline. *p<0.05, **p<0.01 rise from baseline
