# Supplementary material for: Calprotectin (S100A8/A9) has the strongest association with ultrasound-detected synovitis and predicts response to biologic treatment: results from a longitudinal study of patients with established rheumatoid arthritis
Source: Arthritis Res Ther. 2017 Jan 12;19:3. doi: 10.1186/s13075-016-1201-0 (PMC5234113; doi:10.1186/s13075-016-1201-0)
Supplement: Additional file 2: Table S1. — Spearman’s rank correlation coefficients (r s) between biomarkers and US sum scores and clinical parameters during follow-up. (PDF 51 kb) [file 13075_2016_1201_MOESM2_ESM.pdf]

Supplementary table S1. Spearman's rank correlation coefficients (r_s) between biomarkers and sum US scores and clinical parameters during follow-up

	Sum GS score	Sum PD score	Assessor's global VAS	DAS28	Swollen joints (of 32)	Tender joints (of 32)	Patient's global VAS	Joint pain VAS
One month								
**Calprotectin**	0.51**	0.53**	0.51**	0.44**	0.42**	0.21*	0.23*	0.32**
**S100A12**	0.30**	0.38**	0.35**	0.29**	0.28**	0.13	0.08	0.11
**IL-6**	0.39**	0.42**	0.43**	0.38**	0.36**	0.20*	0.15	0.24*
**VEGF**	0.18*	0.17*	-0.03	0.08	-0.01	0.05	0.08	0.17*
**ESR**	0.04	0.14	0.28**	0.56**	0.10	0.08	0.09	0.12
**CRP**	0.21*	0.23*	0.29**	0.30**	0.17*	-0.02	0.05	0.08
Two months								
**Calprotectin**	0.50**	0.53**	0.50**	0.45**	0.46**	0.24*	0.20*	0.25*
**S100A12**	0.26*	0.36**	0.39**	0.32**	0.30**	0.16*	0.19*	0.20*
**IL-6**	0.51**	0.49**	0.55**	0.43**	0.50**	0.25*	0.16	0.20*
**VEGF**	0.21*	0.19*	0.17*	0.11	0.10	0.03	0.09	0.17*
**ESR**	0.06	0.15	0.22*	0.57**	0.09	0.13	0.22*	0.25*
**CRP**	0.30**	0.33**	0.34**	0.30**	0.28**	0.03	0.13	0.13
Three months								
**Calprotectin**	0.37**	0.46**	0.48**	0.44**	0.41**	0.21*	0.29**	0.27**
**S100A12**	0.32**	0.36**	0.36**	0.30**	0.31**	0.18*	0.14	0.12
**IL-6**	0.43**	0.47**	0.53**	0.35**	0.53**	0.18*	0.24*	0.29**
**VEGF**	0.19*	0.16	0.17*	0.03	0.13	-0-07	0.16	0.16
**ESR**	0.01	0.12	0.21*	0.57**	0.05	0.07	0.22*	0.18*
**CRP**	0.18*	0.26*	0.33**	0.44**	0.26**	0.05	0.20*	0.20*
Six months								
**Calprotectin**	0.48**	0.47**	0.44**	0.37**	0.45**	0.12	0.23*	0.20*

**S100A12**	0.26*	0.24*	0.33**	0.27**	0.23*	0.09	0.14	0.18*
**IL-6**	0.40**	0.49**	0.36**	0.36**	0.40**	0.15	0.17*	0.22*
**VEGF**	0.18*	0.12	0.17*	0.04	0.10	0.01	0.14	0.16
**ESR**	0.19*	0.21*	0.22*	0.53**	0.09	0.09	0.09	0.09
**CRP**	0.33**	0.35**	0.35**	0.41**	0.28**	0.07	0.22*	0.17*
Twelve months								
**Calprotectin**	0.25*	0.31**	0.20*	0.25*	0.24*	0.12	0.22*	0.23*
**S100A12**	0.07	0.17*	0.03	0.09	-0.00	-0.04	0.04	0.04
**IL-6**	0.43**	0.43**	0.33**	0.24*	0.37**	0.13	0.11	0.20*
**VEGF**	0.07	0.01	0.02	-0.08	-0.03	-0.08	0.09	0.07
**ESR**	0.01	0.15	0.25*	0.60**	0.11	0.14	0.17*	0.09
**CRP**	0.20*	0.30**	0.27**	0.34**	0.26*	0.14	0.13	0.12

Sum GS score = sum of grey scale scores on a 0-3 scale of 36 joints and four tendon sheaths, sum PD score= sum power Doppler scores on a 0-3 scale of 36 joints and four tendon sheaths, VAS = visual analogue scale, DAS28 = disease activity score of 28 joints (including ESR), VEGF = vascular endothelial growth factor, IL-6 = interleukin 6, ESR = erythrocyte sedimentation rate, CRP = C-reactive protein, *p<0.05, **p≤0.001
