# Supplementary material for: Calprotectin (S100A8/A9) has the strongest association with ultrasound-detected synovitis and predicts response to biologic treatment: results from a longitudinal study of patients with established rheumatoid arthritis
Source: Arthritis Res Ther. 2017 Jan 12;19:3. doi: 10.1186/s13075-016-1201-0 (PMC5234113; doi:10.1186/s13075-016-1201-0)
Supplement: Additional file 3: Table S2. — Spearman’s rank correlation coefficients (r s) between calprotectin and US sum and Lansbury US sum scores. (PDF 33 kb) [file 13075_2016_1201_MOESM3_ESM.pdf]

Supplementary table S2. Spearman's rank correlation coefficients (r_s) between calprotectin and sum US and sum Lansbury US scores

	Sum GS score	Sum LGS score	Sum PD score	Sum LPD score
Calprotectin				
Baseline	0.59**	0.62**	0.62**	0.63**
1 month	0.51**	0.47**	0.53**	0.57**
2 months	0.50**	0.43**	0.53**	0.52**
3 months	0.37**	0.41**	0.46**	0.47**
6 months	0.48**	0.47**	0.47**	0.50**
12 months	0.25*	0.29**	0.31**	0.29**

GS=grey scale, LGS=Lansbury grey scale, PD=power Doppler, LPD=Lansbury power Doppler, *$p<0.01$, **$p\leq0.001$
