# Supplementary material for: Calprotectin (S100A8/A9) has the strongest association with ultrasound-detected synovitis and predicts response to biologic treatment: results from a longitudinal study of patients with established rheumatoid arthritis
Source: Arthritis Res Ther. 2017 Jan 12;19:3. doi: 10.1186/s13075-016-1201-0 (PMC5234113; doi:10.1186/s13075-016-1201-0)
Supplement: Additional file 4: Table S3. — Spearman’s rank correlation coefficients (r s) between calprotectin/conventional inflammatory markers and sum US scores in patients using tocilizumab (n = 12). (PDF 36 kb) [file 13075_2016_1201_MOESM4_ESM.pdf]

Supplementary table S3. Spearman's rank correlation coefficients (r_s) between calprotectin/conventional inflammatory markers and sum ultrasound scores in patients using tocilizumab (n=12)

	Sum GS score						Sum PD score					
	Baseline	1 month	2 months	3 months	6 months	12 months	Baseline	1 month	2 months	3 months	6 months	12 months
Calprotectin	0.66*	0.45	0.53	0.30	0.64*	0.54	0.50	0.27	0.83**	0.67*	0.57	0.57
ESR	-0.007	-0.26	-0.38	-0.27	0.07	0.36	0.21	-0.22	-0.24	0.09	-0.07	0.49
CRP	0.41	0.43	-0.04	-0.11	0.43	0.64*	0.36	0.26	0.22	0.54	0.23	0.72**

Sum GS score = sum of grey scale scores on a 0-3 scale of 36 joints and four tendon sheaths, sum PD score= sum power Doppler scores on a 0-3 scale of 36 joints and four tendon sheaths, ESR =erythrocyte sedimentation rate, CRP = C-reactive protein, *p<0.05, **p≤0.001
