# Supplementary material for: Calprotectin (S100A8/A9) has the strongest association with ultrasound-detected synovitis and predicts response to biologic treatment: results from a longitudinal study of patients with established rheumatoid arthritis
Source: Arthritis Res Ther. 2017 Jan 12;19:3. doi: 10.1186/s13075-016-1201-0 (PMC5234113; doi:10.1186/s13075-016-1201-0)
Supplement: Additional file 5: Figure S2. — Median levels with a range of inflammatory markers in control subjects (n = 100 for calprotectin; n = 141 for S100A12, IL-6 and VEGF) and patients with no PD activity at 12 months (n = 30 for calprotectin, S100A12 and VEGF; n = 28 for IL-6 [patients on tocilizumab omitted]). (PDF 64 kb) [file 13075_2016_1201_MOESM5_ESM.pdf]

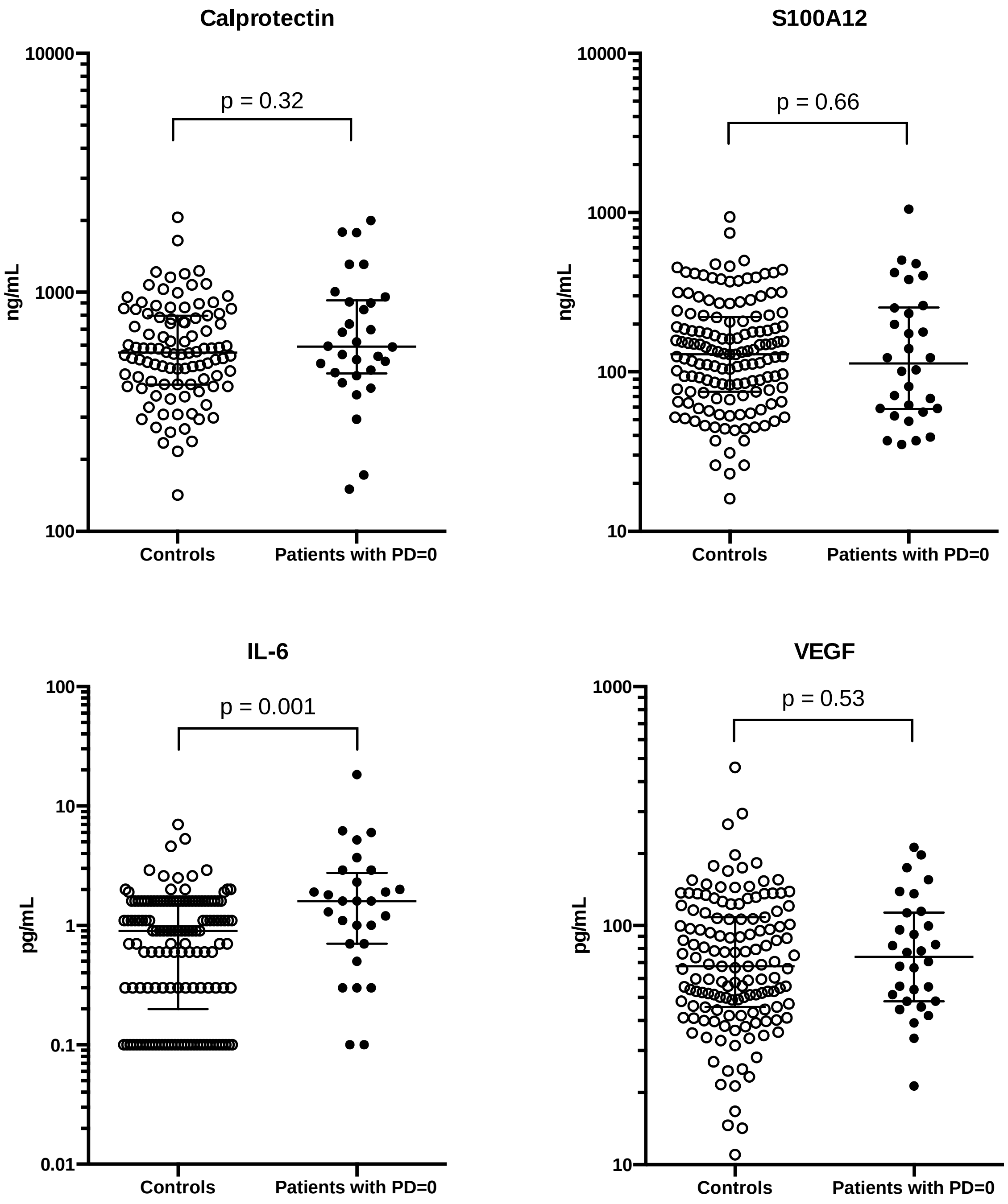

Supplementary figure S2. Median levels with range of inflammatory markers in controls (n=100 for calprotectin, n=141 for S100A12, IL-6 and VEGF) and patients with no PD activity at 12 months (n=30 for calprotectin, S100A12 and VEGF, n=28 for IL-6 (patients on tocilizumab omitted))
