# Supplementary material for: Calprotectin (S100A8/A9) has the strongest association with ultrasound-detected synovitis and predicts response to biologic treatment: results from a longitudinal study of patients with established rheumatoid arthritis
Source: Arthritis Res Ther. 2017 Jan 12;19:3. doi: 10.1186/s13075-016-1201-0 (PMC5234113; doi:10.1186/s13075-016-1201-0)
Supplement: Additional file 6: Table S4. — Spearman’s rank correlation coefficients (r s) between changes in inflammatory markers and US sum scores/DAS28 from baseline to 1, 2, 3, 6 and 12 months. (PDF 40 kb) [file 13075_2016_1201_MOESM6_ESM.pdf]

Supplementary table S4. Spearman's rank correlation coefficients (r_s) between changes in inflammatory markers and sum US scores/DAS28 from baseline to one, two, three, six and 12 months

**Baseline to one month**	**ΔSum GS score**	**ΔSum PD score**	**ΔDAS28**
**ΔCalprotectin**	0.29**	0.35**	0.47**
**ΔS100A12**	0.22*	0.17*	0.22*
**ΔIL-6**	0.10	0.23*	0.15
**ΔVEGF**	0.14	0.11	0.32**
**ΔESR**	0.14	0.20*	0.65**
**ΔCRP**	0.14	0.24*	0.41*
**Baseline to two months**	**ΔSum GS score**	**ΔSum PD score**	**ΔDAS28**
**ΔCalprotectin**	0.39**	0.43**	0.55**
**ΔS100A12**	0.17*	0.18*	0.35**
**ΔIL-6**	0.30**	0.33**	0.31**
**ΔVEGF**	0.12	0.18*	0.26*
**ΔESR**	0.21*	0.30**	0.60**
**ΔCRP**	0.32**	0.33**	0.42**
**Baseline to three months**	**ΔSum GS score**	**ΔSum PD score**	**ΔDAS28**
**ΔCalprotectin**	0.42**	0.46**	0.50**
**ΔS100A12**	0.22*	0.21*	0.31**
**ΔIL-6**	0.25*	0.25*	0.21*
**ΔVEGF**	0.07	0.10	0.22*
**ΔESR**	0.14	0.22*	0.55**
**ΔCRP**	0.20*	0.27**	0.39**
**Baseline to six months**	**ΔSum GS score**	**ΔSum PD score**	**ΔDAS28**
**ΔCalprotectin**	0.43**	0.45**	0.43**
**ΔS100A12**	0.21*	0.23*	0.24*
**ΔIL-6**	0.36**	0.48**	0.28**
**ΔVEGF**	0.07	0.11	0.17*
**ΔESR**	0.24*	0.22*	0.49**
**ΔCRP**	0.34**	0.35**	0.37**
**Baseline to 12 months**	**ΔSum GS score**	**ΔSum PD score**	**ΔDAS28**
**ΔCalprotectin**	0.46**	0.51**	0.46**
**ΔS100A12**	0.27**	0.36**	0.26*
**ΔIL-6**	0.32**	0.38**	0.25*
**ΔVEGF**	0.17*	0.22*	0.25*
**ΔESR**	0.36**	0.42**	0.56**
**ΔCRP**	0.42**	0.50**	0.47**

US = ultrasound scores, DAS28 = disease activity score of 28 joints, GS = grey scale, PD = power Doppler, VEGF = vascular endothelial growth factor, IL-6 = interleukin 6, ESR = erythrocyte sedimentation rate, CRP = C-reactive protein, $*p < 0.05$, $**p \leq 0.001$
