# Supplementary material for: Calprotectin (S100A8/A9) has the strongest association with ultrasound-detected synovitis and predicts response to biologic treatment: results from a longitudinal study of patients with established rheumatoid arthritis
Source: Arthritis Res Ther. 2017 Jan 12;19:3. doi: 10.1186/s13075-016-1201-0 (PMC5234113; doi:10.1186/s13075-016-1201-0)
Supplement: Additional file 7: Table S5. — Median (interquartile range) levels of biomarkers in EULAR good, moderate and non-responder groups at 3, 6 and 12 months. (PDF 39 kb) [file 13075_2016_1201_MOESM7_ESM.pdf]

Supplementary table S5. Median (interquartile range) levels of biomarkers in EULAR good, moderate and non responders at three, six and 12 months

	Good responders	Moderate responders	Non responders	p-value 1	p-value 2	p-value 3	p-value 4
**Three months**	n =44	n=40	n=57				
Calprotectin (ng/mL)	546 (460)	691 (705)	748 (812)	0.04	0.02	0.73	0.11
S100A12 (ng/mL)	110 (248)	192 (234)	154 (216)	0.14	0.08	0.85	0.24
IL-6 (pg/mL)	2.2 (21.1)	3.1 (9.3)	4.5 (8.7)	0.77	0.20	0.36	0.19
VEGF (pg/mL)	78.6 (129.6)	46.6 (97.4)	76.4 (82.9)	0.18	0.87	0.17	0.49
ESR (mm/h)	7 (16)	20 (20)	19 (15)	0.000	0.000	0.87	0.02
CRP (mg/L)	1 (1)	2 (4)	2 (6)	0.02	0.002	0.62	0.03
**Six months**	n=53	n=43	n=45				
Calprotectin (ng/mL)	579 (350)	718 (557)	780 (867)	0.08	0.02	0.34	0.05
S100A12 (ng/mL)	103 (143)	137 (203)	152 (219)	0.17	0.07	0.57	0.15
IL-6 (pg/mL)	1.9 (3.6)	2.4 (4.3)	3.3 (8.5)	0.29	0.02	0.12	0.02
VEGF (pg/mL)	71.9 (90.4)	62.3 (93.7)	76.2 (82.1)	0.98	0.57	0.58	0.52
ESR (mm/h)	10 (12)	18 (16)	12 (14)	0.003	0.03	0.43	0.36
CRP (mg/L)	1 (2)	2 (5)	2 (6)	0.002	0.001	0.71	0.03
**12 months**	n=57	n=41	n=43				
Calprotectin (ng/mL)	619 (486)	641 (502)	666 (834)	0.58	0.22	0.50	0.26
S100A12 (ng/mL)	140 (275)	133 (128)	135 (327)	0.69	0.52	0.29	0.35
IL-6 (pg/mL)	1.9 (3.5)	2.1 (4.0)	2.9 (9.6)	0.82	0.16	0.22	0.13
VEGF (pg/mL)	79.9 (122.8)	68.0 (74.6)	64 (66.8)	0.76	0.58	0.84	0.65
ESR (mm/h)	10 (13)	15 (14)	17 (14)	0.03	0.000	0.25	0.004
CRP (mg/L)	1 (1)	2 (3)	3 (7)	0.23	0.001	0.03	0.001

IL-6 = interleukin 6, VEGF = vascular endothelial growth factor, ESR = erythrocyte sedimentation rate, CRP = C-reactive protein

p-value 1: difference between good and moderate responders, p-value 2: difference between good and non-responders, p-value 3: difference between moderate and non- responders, p-value 4: difference between good/moderate and non-responders
